# Orthodontic Appliance Type and Oral Malodor Burden: Cross-Sectional Comparison of Clear Aligners, Fixed Braces, and Untreated Controls

**DOI:** 10.3390/dj14040225

**Published:** 2026-04-09

**Authors:** Romina Georgiana Bita, Daniel Breban-Schwarzkopf, Magda Mihaela Luca, Edida Maghet, Alexandra Ioana Danila

**Affiliations:** 1 Department II, Radiology and Medical Imaging, General and Dento-Maxillary Imaging, Faculty of Dental Medicine, “Victor Babes” University of Medicine and Pharmacy, Eftimie Murgu Square 2, 300041 Timisoara, Romania; romina.bita@umft.ro; 2 Department of Anatomy and Embryology, “Victor Babes” University of Medicine and Pharmacy, Eftimie Murgu Square 2, 300041 Timisoara, Romania; daniel.breban-schwarzkopf@umft.ro (D.B.-S.); alexandra.danila@umft.ro (A.I.D.); 3 Pediatric Dentistry Research Center (Pedo-Research), Department of Pediatric Dentistry, Faculty of Dental Medicine, “Victor Babes” University of Medicine and Pharmacy, Eftimie Murgu Square 2, 300041 Timisoara, Romania; 4 Faculty of Dental Medicine, “Victor Babes” University of Medicine and Pharmacy, Eftimie Murgu Square 2, 300041 Timisoara, Romania; edida.maghet@umft.ro

**Keywords:** halitosis, orthodontic appliances, clear aligners, fixed orthodontic appliances, dental plaque, tongue coating, salivary flow, oral health-related quality of life

## Abstract

**Background and Objectives:** Halitosis can impair psychosocial well-being, and orthodontic appliances may modify plaque retention and oral ecology. We compared patient-perceived halitosis burden, clinician-rated malodor, and oral health-related quality of life (OHRQoL) among clear aligner users, fixed-brace patients, and untreated controls, and explored oral and salivary correlates of worse malodor severity. **Methods:** This cross-sectional study (March 2024–November 2025) enrolled 184 participants aged 15–35 years (aligners *n* = 62; fixed braces n = 64; controls n = 58). Outcomes were HALT (0–100), organoleptic score (0–5), and OHIP-14 (0–56). Plaque index, gingival inflammation, tongue coating, and unstimulated salivary flow were recorded; low flow was defined as <0.25 mL/min. Organoleptic score ≥ 2 was used descriptively for clinically relevant malodor prevalence, whereas organoleptic score ≥3 defined a moderate-to-severe malodor phenotype for secondary exploratory internal modeling. Multivariable robust linear models (HALT) and proportional-odds ordinal logistic regression (organoleptic severity) were used. **Results:** Fixed braces showed higher HALT (53.7 ± 6.2) than controls (46.3 ± 6.4) and aligners (41.7 ± 7.4) (*p* < 0.001), higher organoleptic scores (2.9 ± 0.4 vs. 2.4 ± 0.6 vs. 2.2 ± 0.6; *p* < 0.001), and worse OHIP-14 (18.6 ± 4.7 vs. 15.9 ± 4.3 vs. 13.8 ± 4.8; *p* < 0.001). Clinically relevant malodor prevalence (organoleptic ≥ 2) was 96.9% in fixed braces, 79.3% in controls, and 66.1% in aligners (*p* < 0.001); because ≥2 was used as a broad descriptive threshold, these values should be interpreted as descriptive rather than diagnostic prevalence estimates. In adjusted models, greater tongue coating, higher plaque, and low salivary flow were associated with worse organoleptic severity, whereas appliance category did not remain independently associated with HALT once concurrent clinical correlates were included. **Conclusions:** Fixed braces showed higher unadjusted malodor burden and worse OHRQoL than aligners and untreated controls, but appliance category should be interpreted as a contextual exposure linked to plaque-retentive conditions rather than as a standalone causal determinant. Plaque accumulation, tongue coating, and lower salivary flow showed the strongest associations with worse malodor severity. These findings should be interpreted in light of the cross-sectional design, possible observer and selection bias, and residual confounding.

## 1. Introduction

Halitosis (oral malodor) is a frequent complaint and remains clinically relevant because it can impair social interactions, self-confidence, and overall well-being. In population-level evidence syntheses, halitosis shows a substantial pooled prevalence across settings, indicating it is not a niche issue restricted to specialty clinics [1]. Beyond prevalence, halitosis is increasingly recognized as a patient-centered condition with measurable psychosocial consequences: systematic review evidence supports a significant association between halitosis and worse oral health-related quality of life (OHRQoL), suggesting that malodor can translate into a meaningful functional and emotional burden for affected individuals [2,3].

Most cases of halitosis are intra-oral and arise from microbial degradation of proteins and peptides with production of volatile compounds, especially volatile sulfur compounds (VSCs). Classic and contemporary literature emphasizes the importance of local ecological drivers such as plaque accumulation, gingival inflammation, and tongue coating in initiating and sustaining malodor [4,5,6]. In support of this biologic framework, quantitative evidence indicates that periodontitis is associated with halitosis, consistent with deeper anaerobic niches and increased VSC production in inflammatory periodontal environments [7,8,9]. Studies have also detected key malodor-related compounds, including hydrogen sulfide and methyl mercaptan, in periodontal pockets, reinforcing the plausibility of periodontal contributions to malodor phenotypes [10,11]. In addition, oral malodor is often described as multifactorial, with salivary hypofunction and xerostomia potentially amplifying odor intensity through reduced clearance, altered buffering, and shifts in microbial homeostasis [8]. For this reason, standardized saliva collection and flow assessment methods are important when investigating halitosis correlates and potential modifiers [10].

A further challenge in halitosis research is measurement heterogeneity. Organoleptic assessment is widely used clinically and captures perceived odor intensity, while device-based approaches (e.g., halitometry) estimate VSC burden; however, meta-analytic findings suggest these methods do not always correlate strongly, implying that reliance on a single metric may incompletely characterize clinical and patient-relevant severity [9]. Accordingly, combining clinician-rated measures (e.g., organoleptic scoring) with patient-reported outcomes can provide a more comprehensive understanding of malodor burden. The Halitosis Associated Life-Quality Test (HALT), including validated language adaptations such as the Romanian version, enables quantification of halitosis-related quality-of-life impact in young adult populations [12].

Orthodontic therapy represents a clinically important context for malodor research because appliances can alter plaque retention, gingival inflammation, and oral hygiene behaviors. Fixed appliances introduce plaque-retentive structures and may increase gingival inflammation, which can plausibly worsen malodor; clinical orthodontic studies have therefore evaluated halitosis alongside periodontal indices during treatment [13]. In contrast, clear aligners are removable, potentially facilitating tooth cleaning and interdental hygiene. Evidence syntheses indicate that aligner therapy is generally associated with better periodontal parameters than fixed appliances, supporting the hypothesis that aligners could be linked to lower malodor burden through improved plaque and gingival control [14,15].

Despite this rationale, comparative studies that simultaneously include untreated controls, integrate both clinician-rated malodor and patient-reported halitosis burden, and evaluate key modifiers such as plaque, tongue coating, and salivary flow in young adults remain limited, particularly in adolescent and young adult populations. Because cross-sectional comparisons across orthodontic treatment modalities may also reflect differences in baseline oral hygiene motivation, treatment selection, and care-seeking behavior, any observed group contrasts should be interpreted as associations rather than causal effects of appliance type alone. Therefore, the present study compares halitosis burden (HALT), organoleptic malodor severity, and OHRQoL (OHIP-14 [15]) across clear aligner users, fixed-braces patients, and controls, and examines how oral indices and salivary measures relate to clinically meaningful malodor phenotypes.

## 2. Materials and Methods

### 2.1. Study Design and Setting

This cross-sectional observational study was conducted to compare oral malodor burden and oral health-related quality of life among clear aligner users, conventional fixed-brace patients, and non-orthodontic controls, with a planned focus on salivary flow as a clinically relevant modifier. The study was implemented in two university-affiliated dental clinics (orthodontics and preventive dentistry) between March 2024 and November 2025. All procedures followed the principles of the Declaration of Helsinki, and participation was voluntary.

Ethics approval was obtained from the institutional review board prior to recruitment. All participants received written and verbal explanations of the study aims, procedures, and confidentiality safeguards. Written informed consent was collected from each adult participant. For participants under 18 years, written consent was obtained from a parent/legal guardian, together with written assent from the participant. Personal identifiers were removed at entry into the study database, and data were stored on a password-protected workstation accessible only to the study team.

### 2.2. Participants, Recruitment, and Grouping

Because orthodontic participants and controls were recruited from different clinical streams, the control group may have differed in care-seeking profile, oral hygiene motivation, and baseline oral health status in ways that could influence plaque, tongue coating, and malodor independently of appliance category. This concern was addressed analytically through adjustment for measured oral hygiene behaviors and clinical oral indices, but residual selection bias and incomplete group comparability cannot be excluded. Participants were recruited consecutively from routine orthodontic follow-up visits (aligner and fixed-brace groups) and from preventive-care appointments (controls). Eligibility criteria were: age 15–35 years, stable general health, and willingness to complete questionnaires and undergo breath and oral assessments. To reduce major confounding, we excluded individuals with: (1) systemic conditions known to markedly affect breath (uncontrolled diabetes, advanced liver/kidney disease), (2) upper respiratory infection in the prior 10 days, (3) antibiotic use in the prior 4 weeks, (4) current daily smoking, and (5) active untreated periodontal disease requiring specialist intervention (screened clinically at the visit). Participants were also excluded if they had used strong antiseptic mouthwash (chlorhexidine) within 24 h, because this could artificially lower malodor measures. To reduce major confounding from advanced periodontal disease, participants were excluded if screening examination showed findings suggestive of active periodontal disease requiring specialist management, including generalized bleeding with visible inflammation, periodontal pocketing judged clinically incompatible with routine preventive or orthodontic follow-up, or other signs prompting referral for periodontal evaluation. Because this was not a dedicated periodontal study, full-mouth periodontal charting was not performed; this is acknowledged as a limitation.

The age range of 15–35 years was selected to capture the adolescent and young adult population most commonly encountered in contemporary orthodontic practice in the participating clinics, while limiting inclusion of older adults with potentially greater age-related periodontal and salivary variability. Age was also retained as a covariate in multivariable analyses to reduce residual confounding.

The study classified participants by current treatment modality (clear aligners versus conventional labial fixed appliances) rather than by individual commercial system or bracket prescription. Detailed information such as aligner brand, attachment design, use of elastics, bracket slot system, ligature type, or prophylaxis schedule was not uniformly available across all participants and was therefore not analyzed. The final analytic sample included 184 participants, distributed into three groups: aligners (n = 62), fixed braces (n = 64), and controls (n = 58). Group definitions were set a priori: (1) aligner users currently wearing removable clear aligners ≥ 3 months, (2) fixed-brace patients currently wearing conventional labial fixed appliances ≥ 3 months, and (3) controls without current orthodontic treatment and without orthodontic treatment in the prior 12 months. Because behavior strongly influences oral malodor, we also recorded pre-specified modifiers for subgroup analyses: frequent snacking (≥3 snack episodes/day vs. fewer), xerostomia symptoms, and salivary flow status (low vs. normal). For participants undergoing orthodontic treatment, treatment duration was defined as the time from treatment initiation to the study enrollment/assessment visit.

A priori sample size planning targeted adequate power to detect a medium difference in HALT scores across three groups (effect size f ≈ 0.28, α = 0.05, power = 0.80), yielding a minimum sample near 168 participants; enrollment was continued to 184 to reduce instability in subgroup analyses (especially low-flow strata).

### 2.3. Measures and Data Collection

All assessments were performed in a standardized morning window (09:00–11:30). Participants were instructed to avoid eating or drinking (water allowed) for 2 h beforehand and to avoid pungent foods/alcohol for 12 h. They were also asked not to brush, floss, chew gum, or use mouthwash during the 2 h pre-assessment window. At the visit, the team recorded demographic data, orthodontic status, and oral hygiene behaviors (brushing frequency, interdental cleaning, tongue cleaning, and mouthwash use). Dietary behavior was simplified into a clinically practical classification: frequent snacking (≥3 episodes/day) vs. infrequent snacking, assessed using a brief standardized interview and a one-page checklist.

Primary outcomes were:

• HALT (Halitosis Associated Life-Quality Test) total score (0–100), assessing perceived halitosis impact.
• Organoleptic scoring was analyzed both as an ordinal clinical severity measure (0–5) and using two pre-specified thresholds with distinct purposes. First, a score of ≥2 was used descriptively to indicate clinically relevant malodor prevalence at the group level. Second, a stricter threshold of ≥3 was used in secondary predictive analyses and figures to represent a moderate-to-severe malodor phenotype. This distinction was adopted to separate broad clinical prevalence estimates from modeling of more pronounced malodor severity. To maintain terminology consistency throughout the manuscript, organoleptic ≥2 is referred to as clinically relevant malodor, whereas organoleptic ≥3 is referred to as a moderate-to-severe malodor phenotype.
• OHIP-14 total score (0–56), reflecting oral health-related quality of life.

To provide context beyond questionnaires alone, we assessed oral conditions that plausibly influence malodor:

• Plaque index using a simplified, standardized 0–3 scale averaged across index tooth sites. Gingival inflammation was also recorded using a simplified gingival index on a 0–3 scale, where higher scores indicated greater visible inflammatory burden during routine clinical examination. This variable was included as an ancillary descriptive oral health measure rather than as a primary predictor in the main multivariable models.
• Tongue coating score on a 0–3 scale (0 = none, 3 = heavy coating), recorded under consistent lighting with the tongue gently protruded.
• Unstimulated whole salivary flow (mL/min) using a practical passive drool technique: participants sat upright and allowed saliva to pool and drip into a pre-weighed container for 5 min. Flow rate was calculated as volume/time. Low flow was defined as <0.25 mL/min, consistent with common clinical thresholds for reduced resting salivary output.

Two examiners completed pre-study calibration sessions to harmonize organoleptic scoring, plaque grading, tongue-coating assessment, and salivary-flow collection instructions. In a subset of participants, duplicate organoleptic scoring was performed for quality control; however, formal inter- and intra-examiner reliability coefficients were not retained for final reporting and are therefore acknowledged as a limitation that may have contributed measurement variability in this subjective outcome.

Complete examiner blinding to treatment group was not feasible because fixed appliances and aligners were clinically visible during assessment. To reduce expectancy bias, organoleptic scoring followed the same pre-specified rubric, assessment sequence, and standardized morning assessment conditions for all groups; however, because masking was not feasible, some observer expectancy cannot be excluded.

Compliance with the pre-assessment instructions was verified on arrival using a brief standardized checklist and verbal confirmation before breath and oral measurements were performed. When participants reported clear protocol deviations that could materially influence breath assessment, the assessment was deferred and rescheduled within the routine clinic workflow.

Height and weight were recorded at the study visit and body mass index (BMI, kg/m^2^) was calculated as weight divided by height squared. BMI was included as a baseline descriptive characteristic because it may relate indirectly to oral health behaviors and dryness-related symptoms.

### 2.4. Statistical Analysis

All analyses were conducted using R statistical software (R Foundation for Statistical Computing, Vienna, Austria; version 4.2) with a two-sided significance threshold of *p* < 0.05. Continuous variables were inspected visually (histograms/Q–Q plots) and formally (Shapiro–Wilk tests) to guide parametric vs. non-parametric testing. For comparisons across the three orthodontic groups, we used one-way ANOVA for normal outcomes (total HALT and OHIP-14) with Tukey post hoc tests for pairwise contrasts. For outcomes with non-normal distributions or clear ordinal behavior (organoleptic scores), we used Kruskal–Wallis tests with appropriate post hoc procedures. Categorical variables (prevalence of organoleptic ≥3, frequent snacking) were compared using chi-square tests, switching to Fisher’s exact test when expected cell counts were small.

To explore patterning beyond basic group comparisons, we pre-specified two additional analytic layers. First, we evaluated whether salivary flow modified appliance-related effects using interaction terms and subgroup analyses (low-flow vs. normal-flow strata). Second, to examine whether a broader clinical feature set improved discrimination of a moderate-to-severe malodor phenotype (organoleptic ≥ 3), we compared a base model with an expanded model including plaque, tongue coating, and salivary flow. These analyses were intended as exploratory, hypothesis-generating internal comparisons rather than as validated clinical prediction tools. Model performance was summarized using the area under the ROC curve (AUC), and incremental performance (ΔAUC) was quantified via bootstrap resampling. Decision-curve analysis (DCA) was used only as an exploratory internal visualization of model behavior across clinically reasonable risk thresholds (0.10–0.50), and not as evidence of clinical readiness, transportability, or external validity.

Finally, associations among key continuous measures (HALT, organoleptic score, OHIP-14, plaque, tongue coating, salivary flow) were examined with Spearman correlation coefficients to avoid over-assuming linearity.

## 3. Results

Across the three groups (aligners, fixed braces, controls), participants were similar in age (22.7–23.1 years; *p* = 0.706), sex distribution (female: 56.2–66.1%; *p* = 0.519), and BMI (22.6–22.9 kg/m^2^; *p* = 0.677), indicating demographic similarity, but not full clinical comparability. As expected, orthodontic treatment duration was longer in the fixed-braces group than in aligners (10.9 ± 2.8 vs. 8.6 ± 2.7 months; *p* < 0.001). Oral hygiene behaviors differed meaningfully: brushing ≥2/day was more frequent in orthodontic patients than controls (88.7% aligners, 85.9% fixed braces vs. 63.8% controls; *p* = 0.001), while daily flossing was highest among aligner users (69.4%) compared with fixed braces (43.8%) and controls (36.2%) (*p* < 0.001). Tongue cleaning was also most common in aligners (62.9% vs. 32.8% fixed braces and 48.3% controls; *p* = 0.003). Mouthwash use was highest in fixed braces (67.2%) compared with aligners (56.5%) and controls (36.2%) (*p* = 0.002), whereas snacking frequency and sugary-snack self-report were similar across groups (*p* = 0.414 and *p* = 0.944). Taken together, the behavioral differences indicate that between-group contrasts should not be interpreted as arising from appliance status alone (Table 1).

Fixed braces showed the least favorable clinical profile, with higher plaque (1.7 ± 0.3) and gingival inflammation (1.7 ± 0.2) compared with aligners (plaque 1.2 ± 0.3; gingival 1.3 ± 0.2) and controls (plaque 1.6 ± 0.6; gingival 1.6 ± 0.3), all with strong between-group differences (*p* < 0.001 for plaque and gingival indices). Tongue coating was also higher in fixed braces (1.4 ± 0.3) than aligners (1.1 ± 0.3) and controls (1.1 ± 0.3) (*p* < 0.001). Unstimulated salivary flow was lowest in fixed braces (0.3 ± 0.1 mL/min) versus 0.4 ± 0.1 mL/min in both aligners and controls (*p* < 0.001). Although the prevalence of objectively “low salivary flow” (<0.25 mL/min) numerically favored aligners (9.7%) over fixed braces (23.4%) and controls (13.8%), this categorical comparison did not reach conventional significance (*p* = 0.094). Xerostomia symptoms, however, were significantly more frequent in fixed braces (42.2%) than aligners (17.7%) and controls (25.9%) (*p* = 0.009), indicating a clinically important subjective dryness burden in the fixed-braces group, as seen in Table 2.

The fixed-braces group had the highest burden across all primary patient-centered outcomes, including HALT total score (53.7 ± 6.2) compared with controls (46.3 ± 6.4) and aligners (41.7 ± 7.4) (*p* < 0.001). Clinician-rated malodor followed the same gradient (organoleptic 2.9 ± 0.4 fixed braces vs. 2.4 ± 0.6 controls vs. 2.2 ± 0.6 aligners; *p* < 0.001), alongside worse oral health-related quality of life (OHIP-14: 18.6 ± 4.7 fixed braces vs. 15.9 ± 4.3 controls vs. 13.8 ± 4.8 aligners; *p* < 0.001). Using the descriptive threshold of organoleptic ≥2, clinically relevant malodor prevalence was 96.9% in fixed braces, 79.3% in controls, and 66.1% in aligners (*p* < 0.001), as seen in Table 3. Because this low threshold may capture mild or borderline odor findings and is lower than cutoffs used in some prior reports, these percentages should be interpreted as broad descriptive indicators of detectable malodor burden within this clinic-based cohort rather than as diagnostic prevalence estimates of established halitosis [16,17]. Post hoc testing confirmed a consistent gradient across outcomes, but these between-group differences should be interpreted with caution because recruitment structure and oral hygiene differences may also have contributed.

Within both salivary-flow strata, fixed braces consistently showed worse outcomes than aligners and controls. Among participants with low flow, fixed braces had higher HALT (57.1 ± 5.9) than aligners (45.6 ± 6.7) and controls (48.4 ± 3.6) (*p* < 0.001), higher organoleptic scores (3.1 ± 0.3 vs. 2.4 ± 0.4 vs. 2.7 ± 0.4; *p* = 0.003), and higher OHIP (20.4 ± 4.3 vs. 14.7 ± 1.9 vs. 16.9 ± 4.6; *p* = 0.018). The same pattern held in normal-flow participants (HALT: 52.6 ± 5.9 fixed braces vs. 41.3 ± 7.4 aligners vs. 45.9 ± 6.7 controls; *p* < 0.001; organoleptic: 2.9 ± 0.4 vs. 2.2 ± 0.6 vs. 2.4 ± 0.6; *p* < 0.001; OHIP: 18.1 ± 4.7 vs. 13.7 ± 5.1 vs. 15.8 ± 4.2; *p* < 0.001). Importantly, the group×flow interaction was not significant for HALT (0.81), organoleptic (0.952), or OHIP (0.816), indicating that salivary flow did not meaningfully modify the relative differences between orthodontic groups, even though low flow tended to coincide with worse absolute scores (Table 4).

Frequent snacking was associated with higher absolute symptom burden, but the orthodontic-group pattern remained consistent regardless of snacking status. In frequent snackers, fixed braces had the highest HALT (55.4 ± 5.4) compared with controls (47.6 ± 6.7) and aligners (44.8 ± 7.2) (*p* < 0.001), as well as higher organoleptic scores (3.1 ± 0.3 vs. 2.4 ± 0.6 vs. 2.3 ± 0.6; *p* < 0.001) and OHIP (18.9 ± 3.8 vs. 16.1 ± 4.8 vs. 14.4 ± 4.4; *p* < 0.001). In infrequent snackers, the same hierarchy persisted (HALT: 52.1 ± 6.4 fixed braces vs. 45.4 ± 6.1 controls vs. 38.8 ± 6.3 aligners; *p* < 0.001; organoleptic: 2.8 ± 0.4 vs. 2.4 ± 0.6 vs. 2.1 ± 0.6; *p* < 0.001; OHIP: 18.4 ± 5.4 vs. 15.9 ± 3.9 vs. 13.3 ± 5.2; *p* < 0.001). Plaque index differences mirrored clinical risk (frequent snackers: 1.8 ± 0.3 fixed braces vs. 1.6 ± 0.6 controls vs. 1.3 ± 0.3 aligners; *p* < 0.001), and interaction terms were non-significant (group×snacking: HALT 0.237; organoleptic 0.181; OHIP 0.848; plaque 0.514), suggesting snacking does not substantially change the between-group contrasts (Table 5).

Bivariate associations showed that patient-reported halitosis burden (HALT) aligned most strongly with clinician-rated malodor (ρ = 0.6, *p* < 0.001), indicating substantial concordance between subjective impact and clinical assessment. HALT also correlated moderately with OHIP (ρ = 0.4, *p* < 0.001), plaque (ρ = 0.4, *p* < 0.001), and tongue coating (ρ = 0.4, *p* < 0.001), supporting a coherent pattern linking malodor-related quality-of-life impairment to oral biofilm and tongue findings. Salivary flow correlated inversely with HALT (ρ = −0.3, *p* < 0.001), organoleptic (ρ = −0.3, *p* < 0.001), and OHIP (ρ = −0.3, *p* < 0.001), consistent with dryness as a contributor to worse breath and QoL. Snacking frequency had smaller positive correlations with HALT (ρ = 0.2, *p* < 0.01), organoleptic (ρ = 0.2, *p* < 0.05), and tongue coating (ρ = 0.2, *p* < 0.05), suggesting a weaker but detectable behavioral component (Table 6).

After multivariable adjustment, the strongest independent predictor of HALT was the organoleptic score: each +1 increase was associated with a +6.2-point higher HALT (95% CI 4.3 to 8.0; *p* < 0.001; partial R^2^ 20.2%). Tongue coating was also a major driver (+5.1 per +1; 95% CI 2.4 to 7.8; *p* < 0.001; partial R^2^ 7.6%), as was OHIP-14 (+0.3 per point; 95% CI 0.1 to 0.5; *p* < 0.001; partial R^2^ 7.2%). Xerostomia symptoms independently added a clinically meaningful increment (+2.4; 95% CI 0.6 to 4.1; *p* = 0.008; partial R^2^ 4.0%). In contrast, being in the fixed-braces group versus aligners was not statistically significant once these clinical and behavioral factors were included (β = 2.2; *p* = 0.101), and the fixed braces×low-flow interaction was also non-significant (β = 0.9; *p* = 0.663), consistent with the stratified analyses. Overall fit was high (R^2^ = 0.8; adjusted R^2^ = 0.7; global *p* < 0.001), and multicollinearity appeared acceptable (VIF range 1.1–2.6). This pattern suggests that the higher self-reported burden observed in fixed-brace patients was largely accounted for by concurrent clinical severity and symptom correlates rather than appliance category alone (Table 7).

Greater plaque and tongue coating were the strongest predictors of worse organoleptic severity: each +1 increase in plaque index carried an OR of 4.3 (95% CI 2.1–9.1; *p* < 0.001), while each +1 increase in tongue coating index carried an OR of 9.9 (95% CI 3.7–26.3; *p* < 0.001), indicating a very steep escalation in the odds of being in a higher malodor category with tongue biofilm. Low salivary flow also increased severity (OR 2.5; 95% CI 1.1–5.9; *p* = 0.036). Fixed braces remained an independent risk factor versus aligners even after adjustment (OR 4.9; 95% CI 1.9–12.5; *p* < 0.001), whereas controls did not differ from aligners (OR 1.1; *p* = 0.764). Female sex showed a modest but significant association with higher severity (OR 1.9; 95% CI 1.0–3.5; *p* = 0.034), while snacking, flossing, tongue cleaning, and age were not significant in this model. Thus, appliance category appears more closely linked to clinician-rated malodor severity than to self-reported halitosis burden once other concurrent clinical correlates are considered (Table 8).

**Figure 1:**
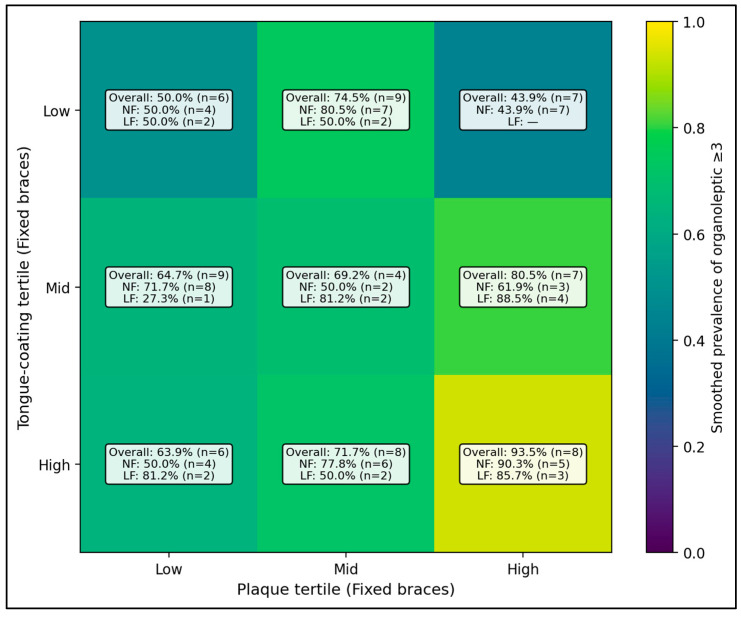
Fixed-braces “risk phenotype” heatmap (Plaque tertile × Tongue-coating tertile).

Among patients with fixed braces, the heatmap shows that the chance of more noticeable bad breath (organoleptic ≥ 3) rises sharply when both plaque and tongue coating are high. The highest-risk pattern is the high plaque + high tongue coating cell, where 93.5% of patients met the ≥3 threshold (n = 8). In contrast, when tongue coating is low and plaque is low-to-mid, the proportion with organoleptic ≥3 stays much lower (for example, 36.6% (n = 7) in the low tongue + low plaque cell). When the same cells are split by salivary flow, the pattern is generally similar, but some “high plaque/high tongue” combinations remain high even when flow is normal, suggesting that the combination of plaque + tongue coating is a strong driver of worse breath in fixed-brace patients (Figure 1).

**Figure 2:**
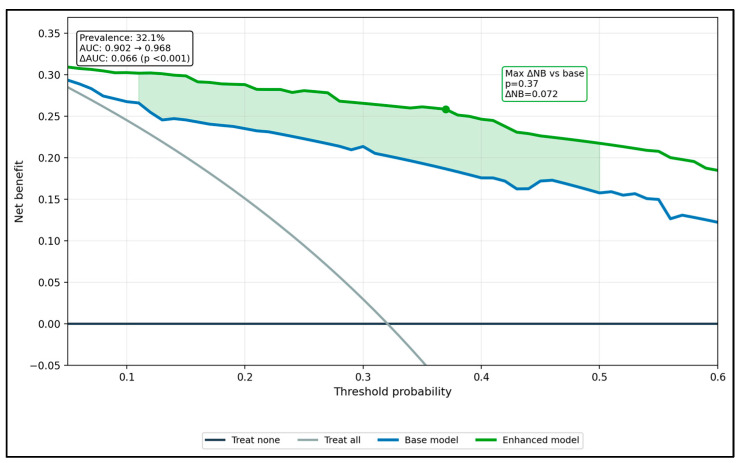
Decision-curve analysis (DCA) for predicting organoleptic ≥ 3.

In exploratory internal analyses, the expanded model showed higher apparent discrimination than the simpler model (AUC 0.902 vs. 0.968). However, because model development, bootstrap estimation, and decision-curve analysis were all performed within the same modest dataset, with some small subgroup strata, these estimates are likely optimism-prone and should be regarded only as exploratory internal performance summaries rather than as evidence of transportable or clinically ready prediction models (Figure 2).

## 4. Discussion

### 4.1. Analysis of Findings

In this cohort of adolescents and young adults, fixed braces showed the most unfavorable malodor phenotype across both clinician-rated (organoleptic) and patient-centered outcomes (HALT, OHIP-14), while aligners consistently showed the lowest burden and controls were intermediate. This gradient is broadly compatible with prospective orthodontic evidence suggesting that fixed appliances can acutely increase plaque/gingival indices and raise oral malodor shortly after bonding, with malodor reaching clinically relevant levels in some patients [18]. Longer follow-up data also indicate that malodor-related measures can remain elevated across treatment milestones in fixed-appliance cohorts [16,17,19,20], although the overall evidence base remains heterogeneous and, at the systematic-review level, is often limited by small trials, inconsistent malodor definitions, and variable cutoffs for halitosis [16]. In that context, the present study adds value by including an untreated control group and by pairing clinician-rated malodor with patient-perceived burden (HALT) and OHRQoL in the same analytic framework. At the same time, the very high prevalence estimates obtained with organoleptic ≥2 should be interpreted against the intentionally descriptive nature of this low threshold and the broader literature showing substantial heterogeneity in malodor cutoffs and assessment methods [16,17]. Accordingly, these percentages are better understood as broad indicators of detectable or borderline malodor burden in this cohort than as diagnostic prevalence estimates of established halitosis.

The apparent difference between multivariable models reflects the fact that they estimate different outcomes. In the HALT model, appliance group is evaluated after accounting for concurrent clinician-rated malodor, OHRQoL, tongue coating, and xerostomia, so its residual association is attenuated and no longer statistically significant. By contrast, the ordinal logistic model evaluates clinician-rated malodor severity directly, where fixed braces remained independently associated with worse categories. Accordingly, appliance type is better interpreted here as a contextual exposure linked to plaque-retentive conditions and related clinical correlates, rather than as the sole independent determinant of halitosis burden across all outcome domains.

One finding that warrants specific comment is that aligner users had lower malodor burden than untreated controls. This should not be interpreted as aligners being intrinsically protective. Rather, in this cohort, the aligner group also demonstrated more favorable oral hygiene behaviors, particularly daily flossing and tongue cleaning, and the removability of aligners may have facilitated more effective hygiene around teeth and gingival margins. At the same time, controls were recruited from a preventive-care stream rather than from the same orthodontic follow-up pathway, so the aligner-versus-control contrast may also reflect differences in care-seeking behavior, hygiene motivation, and baseline oral health profile rather than a pure treatment effect.

Mechanistically, our multivariable and ordinal models converge on a coherent biological explanation: fixed braces appear to increase plaque retention and tongue biofilm, and these two substrates strongly track with worse organoleptic severity (with tongue coating showing the steepest risk gradient). This pattern is highly consistent with classic VSC-pathway evidence in which periodontal inflammatory substrates and tongue coating contribute to oral malodor chemistry. In periodontal patients, Yaegaki and Sanada reported that clinical/biochemical factors including tongue coating and gingival fluid enhance VSC production [21,22]. Likewise, population-level data from Miyazaki and colleagues found that VSC values correlate significantly with periodontal conditions and tongue coating status, supporting tongue/periodontal niches as key determinants of mouth-air malodor [23]. Importantly, this helps interpret why fixed braces remained independently associated with worse organoleptic categories even after adjustment: fixed appliances plausibly promote both the microbial biomass and the ecological niches (around brackets and in inflamed gingiva) that supply VSC precursors, while also making hygiene more technically challenging despite high reported brushing frequency.

A particularly actionable finding is the magnitude of the tongue-coating signal: in our cohort, tongue coating was not a minor correlate but a strong predictor of clinically meaningful malodor (organoleptic ≥ 3) and a major independent driver of HALT burden. This aligns with clinical observational evidence that tongue coating is itself patterned by hygiene ecology: Van Tornout et al. reported that oral hygiene level is the strongest determinant of tongue coating presence in malodor-complaining populations, with other contributors (e.g., periodontal status, dietary habits) playing smaller roles [24,25]. In orthodontic care pathways, tongue status is often under-emphasized relative to bracket-associated plaque control; our results suggest this is a missed opportunity, particularly for fixed-brace patients, where “high plaque + high tongue coating” co-occurred with extremely high rates of moderate malodor. Thus, beyond reinforcing standard orthodontic hygiene counseling, the present data support explicitly integrating tongue-coating assessment and technique-specific tongue cleaning into routine follow-up—especially because tongue coating retained strong effects even when self-reported “tongue cleaning” as a behavior did not independently predict outcomes after adjustment (likely reflecting large variation in technique quality and adherence).

Salivary factors also fit plausibly into the model, but with an important nuance: fixed braces showed lower unstimulated flow and markedly higher xerostomia symptoms, and flow correlated inversely with HALT, organoleptic, and OHIP-14. Yet, the non-significant group×flow interaction indicates that reduced flow is better interpreted as an additive risk factor rather than the primary explanation for the appliance-related gradient. This is consistent with clinical physiology evidence that reduced salivary output is associated with greater VSC burden and worse malodor biochemistry. In a clinical study using gas chromatography to quantify key VSCs, Koshimune et al. specifically evaluated low salivary flow and volatile sulfur compounds, supporting the concept that salivary hypofunction can amplify malodor risk [26]. On the other hand, not all orthodontic modalities appear to induce dryness or malodor uniformly: Schaefer et al. reported that aligner (Invisalign) treatment, in their cohort, did not cause halitosis or oral dryness and had minimal overall impairment, which is directionally consistent with our “aligners as lowest-burden phenotype” finding [21]. This aligns with the broader orthodontic-halitosis evidence synthesis, which highlights both the uncertainty caused by inconsistent diagnostic cutoffs and the need for better controlled trials with standardized, multi-domain endpoints (organoleptic/VSC + patient-reported impact) [16]. A logical next step is therefore longitudinal, pre–post appliance studies (with controls) that measure tongue-coating dynamics, salivary flow, and malodor outcomes at standardized times of day—paired with randomized preventive bundles (enhanced interproximal cleaning, structured tongue-cleaning training, and targeted anti-VSC rinses) to determine what most effectively reduces clinically relevant malodor and, critically, improves HALT and OHRQoL in routine orthodontic practice.

These findings may have practical relevance for orthodontic follow-up, particularly by highlighting plaque control, tongue coating, and xerostomia symptoms as clinically observable correlates of worse malodor. However, because the study is cross-sectional and susceptible to selection and measurement bias, the data should not be used alone to recommend one orthodontic modality over another. Instead, the results may help inform hypothesis-driven prevention strategies to be tested prospectively. In particular, the observed group differences likely reflect a combination of appliance-related plaque retention, oral hygiene behavior, recruitment-channel differences, and possible observer effects, so appliance type should not be over-interpreted as a standalone causal factor. Nevertheless, these findings should be interpreted within the specific context of the study, as patient characteristics, demographic variability, clinical heterogeneity, comorbidity burden, and healthcare-setting factors may influence both the observed associations and the broader applicability of the results [27,28,29,30,31,32,33,34,35,36,37,38].

### 4.2. Study Limitations

The cross-sectional design limits causal inference and cannot determine whether appliance type caused changes in malodor over time. Participants were recruited from university-affiliated clinics, which may reduce generalizability to broader community settings and different age distributions. Oral hygiene behaviors and diet (snacking) were partly self-reported, introducing recall and social-desirability bias, and technique quality (tongue cleaning) could not be fully standardized. Organoleptic assessment remains subjective compared with gas chromatography or standardized VSC devices, and chemical breath profiling was not incorporated. Recruitment of orthodontic groups from orthodontic follow-up clinics and controls from preventive-care visits is an important potential source of selection bias, because these streams may differ in motivation, oral health awareness, and baseline oral conditions in ways that influence plaque, tongue coating, and malodor independently of appliance type. In addition, organoleptic assessment was not fully blinded to orthodontic status because appliances were visible; although standardized conditions and pre-study calibration were used, observer expectancy may have influenced assignment of adjacent severity categories and potentially the magnitude of between-group differences. Formal inter- and intra-examiner reliability coefficients for repeated organoleptic scoring were not retained for reporting, which limits our ability to distinguish true between-group variation from measurement variability in this subjective outcome. Residual confounding remains possible (e.g., oral microbiome differences, subtle respiratory contributors, adherence variability), some subgroup analyses were based on small cell sizes and are statistically fragile, and we did not stratify orthodontic treatment according to aligner system, attachment complexity, bracket design, ligature type, elastics use, or prophylaxis schedule.

## 5. Conclusions

In this cohort of adolescents and young adults, fixed braces showed higher unadjusted halitosis-related burden and worse clinician-rated malodor than aligners and untreated controls. Tongue coating and plaque accumulation showed the strongest associations with worse malodor severity, while reduced salivary flow appeared to add risk. However, appliance category did not remain independently associated with HALT after adjustment for concurrent clinical correlates, and the observed group differences likely reflect a combination of plaque-retentive conditions, oral hygiene behavior, recruitment structure, and possible measurement bias. These findings should therefore be viewed as hypothesis-generating and warrant prospective confirmation.

## Figures and Tables

**Table 1:** Baseline characteristics and oral hygiene behaviors.

Characteristic	Aligners (n = 62)	Fixed Braces (n = 64)	Controls (n = 58)	*p* Value
Age, years	22.9 ± 2.6	23.1 ± 2.3	22.7 ± 2.2	0.706
Female sex	41 (66.1)	36 (56.2)	36 (62.1)	0.519
BMI, kg/m^2^	22.6 ± 2.8	22.9 ± 2.8	22.9 ± 2.6	0.677
Orthodontic treatment duration, months	8.6 ± 2.7	10.9 ± 2.8	—	<0.001
Brushing ≥ 2/day	55 (88.7)	55 (85.9)	37 (63.8)	0.001
Daily flossing	43 (69.4)	28 (43.8)	21 (36.2)	<0.001
Daily tongue cleaning	39 (62.9)	21 (32.8)	28 (48.3)	0.003
Daily mouthwash use	35 (56.5)	43 (67.2)	21 (36.2)	0.002
Frequent snacking (≥3/day)	30 (48.4)	31 (48.4)	22 (37.9)	0.414
Sugary snacks (self-report)	25 (40.3)	26 (40.6)	25 (43.1)	0.944

Tests: ANOVA (age, BMI), Welch *t*-test (treatment duration), χ^2^ (categoricals). Continuous shown as mean ± SD; categorical as n (%); BMI, body mass index; n, number.

**Table 2:** Clinical oral indices and salivary measures.

Clinical Measure	Aligners (n = 62)	Fixed Braces (n = 64)	Controls (n = 58)	*p* Value
Plaque index (0–3)	1.2 ± 0.3	1.7 ± 0.3	1.6 ± 0.6	<0.001
Gingival index (0–3)	1.3 ± 0.2	1.7 ± 0.2	1.6 ± 0.3	<0.001
Tongue coating index (0–3)	1.1 ± 0.3	1.4 ± 0.3	1.1 ± 0.3	<0.001
Unstimulated salivary flow, mL/min	0.4 ± 0.1	0.3 ± 0.1	0.4 ± 0.1	<0.001
Low salivary flow (<0.25 mL/min)	6 (9.7)	15 (23.4)	8 (13.8)	0.094
Xerostomia symptoms (yes)	11 (17.7)	27 (42.2)	15 (25.9)	0.009

**Table 3:** Primary outcomes by orthodontic status.

Outcome	Aligners (n = 62)	Fixed Braces (n = 64)	Controls (n = 58)	*p* Value
HALT total score (0–100)	41.7 ± 7.4	53.7 ± 6.2	46.3 ± 6.4	<0.001
Organoleptic score (0–5)	2.2 ± 0.6	2.9 ± 0.4	2.4 ± 0.6	<0.001
OHIP-14 total score (0–56)	13.8 ± 4.8	18.6 ± 4.7	15.9 ± 4.3	<0.001
Clinically relevant malodor prevalence (organoleptic ≥2)	41 (66.1)	62 (96.9)	46 (79.3)	<0.001

Post hoc Tukey HSD (HALT, OHIP-14) showed all pairwise differences as significant (*p* ≤ 0.036). Organoleptic compared via Kruskal–Wallis; HALT, Halitosis Associated Life-Quality Test; OHIP-14, Oral Health Impact Profile-14.

**Table 4:** Outcomes stratified by salivary flow status.

Outcome	Aligners Low Flow	Fixed Braces Low Flow	Controls Low Flow	*p* (Group|Low)	Aligners Normal Flow	Fixed Braces Normal Flow	Controls Normal Flow	*p* (Group|Normal)	*p* (Group×Flow)
HALT	6; 45.6 ± 6.7	15; 57.1 ± 5.9	8; 48.4 ± 3.6	<0.001	56; 41.3 ± 7.4	49; 52.6 ± 5.9	50; 45.9 ± 6.7	<0.001	0.81
organoleptic	6; 2.4 ± 0.4	15; 3.1 ± 0.3	8; 2.7 ± 0.4	0.003	56; 2.2 ± 0.6	49; 2.9 ± 0.4	50; 2.4 ± 0.6	<0.001	0.952
OHIP	6; 14.7 ± 1.9	15; 20.4 ± 4.3	8; 16.9 ± 4.6	0.018	56; 13.7 ± 5.1	49; 18.1 ± 4.7	50; 15.8 ± 4.2	<0.001	0.816

Tests: Within-stratum. Interaction *p* from two-way ANOVA; HALT, Halitosis Associated Life-Quality Test; OHIP-14, Oral Health Impact Profile-14.

**Table 5:** Outcomes stratified by snacking frequency.

Outcome	Aligners Frequent Snacking	Fixed Braces Frequent Snacking	Controls Frequent Snacking	*p* (Group|Frequent)	Aligners Infrequent Snacking	Fixed Braces Infrequent Snacking	Controls Infrequent Snacking	*p* (Group|Infrequent)	*p* (Group×Snacking)
HALT	30; 44.8 ± 7.2	31; 55.4 ± 5.4	22; 47.6 ± 6.7	<0.001	32; 38.8 ± 6.3	33; 52.1 ± 6.4	36; 45.4 ± 6.1	<0.001	0.237
organoleptic	30; 2.3 ± 0.6	31; 3.1 ± 0.3	22; 2.4 ± 0.6	<0.001	32; 2.1 ± 0.6	33; 2.8 ± 0.4	36; 2.4 ± 0.6	<0.001	0.181
OHIP	30; 14.4 ± 4.4	31; 18.9 ± 3.8	22; 16.1 ± 4.8	<0.001	32; 13.3 ± 5.2	33; 18.4 ± 5.4	36; 15.9 ± 3.9	<0.001	0.848
plaque	30; 1.3 ± 0.3	31; 1.8 ± 0.3	22; 1.6 ± 0.6	<0.001	32; 1.2 ± 0.4	33; 1.6 ± 0.3	36; 1.6 ± 0.6	<0.001	0.514

Tests: Within-stratum. Interaction *p* from two-way ANOVA; HALT, Halitosis Associated Life-Quality Test; OHIP-14, Oral Health Impact Profile-14.

**Table 6:** Spearman correlation matrix.

	HALT	Organoleptic	OHIP	Plaque	Tongue_Coat	Sal_Flow	Snack_Freq
HALT	—						
organoleptic	0.6 ***	—					
OHIP	0.4 ***	0.4 ***	—				
plaque	0.4 ***	0.3 ***	0.2 **	—			
tongue_coat	0.4 ***	0.4 ***	0.2 **	0.2 **	—		
sal_flow	−0.3 ***	−0.3 ***	−0.3 ***	−0.2 **	−0.2 *	—	
snack_freq	0.2 **	0.2 *	0.1	0.1	0.2 *	−0.1	—

Significance: * *p* < 0.05, ** *p* < 0.01, *** *p* < 0.001; HALT, Halitosis Associated Life-Quality Test; OHIP-14, Oral Health Impact Profile-14.

**Table 7:** Multivariable model of HALT burden.

Predictor	β (HC3 SE)	95% CI	*p*	VIF	Partial R^2^ (%)
Fixed braces (vs. aligners)	2.2 (1.3)	−0.4 to 4.8	0.101	2.6	1.6
Controls (vs. aligners)	1.8 (1.1)	−0.3 to 4.0	0.095	1.9	1.6
Organoleptic score (per +1)	6.2 (0.9)	4.3 to 8.0	<0.001	2.6	20.2
OHIP-14 total (per +1)	0.3 (0.1)	0.1 to 0.5	<0.001	1.6	7.2
Plaque index (per +1)	0.7 (0.9)	−1.0 to 2.5	0.415	1.7	0.4
Tongue coating index (per +1)	5.1 (1.4)	2.4 to 7.8	<0.001	1.6	7.6
Low salivary flow (<0.25 mL/min)	1.3 (1.6)	−1.8 to 4.4	0.397	2.3	0.4
Fixed braces × low flow (interaction)	0.9 (2.0)	−3.1 to 4.9	0.663	2.5	0.1
Frequent snacking (≥3/day)	0.4 (0.8)	−1.1 to 1.9	0.609	1.1	0.2
Daily flossing	0.3 (0.7)	−1.2 to 1.8	0.699	1.3	0.1
Daily tongue cleaning	1.0 (0.8)	−0.6 to 2.6	0.217	1.2	0.9
Xerostomia symptoms	2.4 (0.9)	0.6 to 4.1	0.008	1.3	4
Age (per year)	−0.1 (0.2)	−0.4 to 0.2	0.550	1.1	0.2
Female sex	0.3 (0.8)	−1.3 to 1.8	0.741	1.1	0.1

Outcome: HALT total score (0–100). Model: Robust OLS with HC3 standard errors. Reference group = aligners. Model fit: R^2^ = 0.8, adjusted R^2^ = 0.7; global model *p* < 0.001; β, regression coefficient; SE, standard error; HC3, heteroskedasticity-consistent standard errors (type 3); CI, confidence interval; VIF, variance inflation factor; OLS, ordinary least squares; R^2^, coefficient of determination.

**Table 8:** Ordinal (proportional-odds) model for organoleptic severity phenotype.

Predictor	OR (95% CI)	β (SE)	*p*
Plaque index (per +1)	4.3 (2.1–9.1)	1.5 (0.4)	<0.001
Tongue coating index (per +1)	9.9 (3.7–26.3)	2.3 (0.5)	<0.001
Low salivary flow (<0.25 mL/min)	2.5 (1.1–5.9)	0.9 (0.4)	0.036
Frequent snacking (≥3/day)	1.0 (0.5–1.8)	−0.0 (0.3)	0.89
Daily tongue cleaning	1.1 (0.6–2.1)	0.1 (0.3)	0.684
Daily flossing	0.7 (0.4–1.4)	−0.3 (0.3)	0.334
Age (per year)	1.0 (0.9–1.1)	−0.0 (0.1)	0.613
Female sex	1.9 (1.0–3.5)	0.7 (0.3)	0.034
Controls (vs. aligners)	1.1 (0.5–2.5)	0.1 (0.4)	0.764
Fixed braces (vs. aligners)	4.9 (1.9–12.5)	1.6 (0.5)	<0.001

Model: Proportional-odds ordinal logistic regression. Interpretation: OR > 1 = higher odds of being in a worse organoleptic category; OR, odds ratio; β, regression coefficient; SE, standard error; CI, confidence interval.

## Data Availability

The data presented in this study are available on request from the corresponding author. The data are not publicly available due to privacy and ethical restrictions.

## References

[1] Silva M.F., Leite F.R.M., Ferreira L.B., Pola N.M., Scannapieco F.A., Demarco F.F., Nascimento G.G. (2018). Estimated prevalence of halitosis: A systematic review and meta-regression analysis. Clin. Oral Investig..

[2] Schertel Cassiano L., Abdullahi F., Leite F.R.M., López R., Peres M.A., Nascimento G.G. (2021). The association between halitosis and oral-health-related quality of life: A systematic review and meta-analysis. J. Clin. Periodontol..

[3] Zalewska A., Zatoński M., Jabłonka-Strom A., Paradowska A., Kawala B., Litwin A. (2012). Halitosis—A common medical and social problem. A review on pathology, diagnosis and treatment. Acta Gastroenterol. Belg..

[4] van den Broek A.M., Feenstra L., de Baat C. (2007). A review of the current literature on aetiology and measurement methods of halitosis. J. Dent..

[5] Kaygisiz E., Uzuner F.D., Yuksel S., Taner L., Çulhaoğlu R., Sezgin Y., Ateş C. (2015). Effects of self-ligating and conventional brackets on halitosis and periodontal conditions. Angle Orthod..

[6] Uzuner F.D., Kaygisiz E., Cankaya Z.T. (2014). Effect of the bracket types on microbial colonization and periodontal status. Angle Orthod..

[7] Silva M.F., Cademartori M.G., Leite F.R.M., López R., Demarco F.F., Nascimento G.G. (2017). Is periodontitis associated with halitosis? A systematic review and meta-regression analysis. J. Clin. Periodontol..

[8] Silveira J.O.D., Cota L.O.M., Bendo C.B., Faria S.F.S., Costa F.O. (2020). Validation of the Brazilian version of the Halitosis Associated Life-Quality Test (HALT). Braz. Oral Res..

[9] Szalai E., Tajti P., Szabó B., Kói T., Hegyi P., Czumbel L.M., Varga G., Kerémi B. (2023). Organoleptic and Halitometric Assessments Do Not Correlate Well in Intra-Oral Halitosis: A Systematic Review and Meta-Analysis. J. Evid. Based Dent. Pract..

[10] Navazesh M. (1993). Methods for collecting saliva. Ann. N. Y. Acad. Sci..

[11] Cardoso Mde A., Saraiva P.P., Maltagliati L.Á., Rhoden F.K., Costa C.C., Normando D., Capelozza Filho L. (2015). Alterations in plaque accumulation and gingival inflammation promoted by treatment with self-ligating and conventional orthodontic brackets. Dent. Press J. Orthod..

[12] Briceag R., Caraiane A., Raftu G., Bratu M.L., Buzatu R., Dehelean L., Bondrescu M., Bratosin F., Bumbu B.A. (2023). Validation of the Romanian Version of the Halitosis Associated Life-Quality Test (HALT) in a Cross-Sectional Study among Young Adults. Healthcare.

[13] Murakami S., Mealey B.L., Mariotti A., Chapple I.L.C. (2018). Dental plaque-induced gingival conditions. J. Periodontol..

[14] Rossini G., Parrini S., Castroflorio T., Deregibus A., Debernardi C.L. (2015). Periodontal health during clear aligners treatment: A systematic review. Eur. J. Orthod..

[15] Slade G.D. (1997). Derivation and validation of a short-form oral health impact profile. Community Dent. Oral Epidemiol..

[16] Abdulraheem S., Paulsson L., Petrén S., Sonesson M. (2019). Do fixed orthodontic appliances cause halitosis? A systematic review. BMC Oral Health.

[17] Huang J., Li C.Y., Jiang J.H. (2018). Effects of fixed orthodontic brackets on oral malodor: A systematic review and meta-analysis according to the preferred reporting items for systematic reviews and meta-analyses guidelines. Medicine.

[18] Babacan H., Sokucu O., Marakoglu I., Ozdemir H., Nalcaci R. (2011). Effect of fixed appliances on oral malodor. Am. J. Orthod. Dentofac. Orthop..

[19] Sökücü O., Akpınar A., Özdemir H., Birlik M., Çalışır M. (2016). The effect of fixed appliances on oral malodor from beginning of treatment till 1 year. BMC Oral Health.

[20] Zurfluh M.A., van Waes H.J., Filippi A. (2013). The influence of fixed orthodontic appliances on halitosis. Schweiz. Monatsschr. Zahnmed..

[21] Levrini L., Mangano A., Montanari P., Margherini S., Caprioglio A., Abbate G.M. (2015). Periodontal health status in patients treated with the Invisalign^®^ system and fixed orthodontic appliances: A 3 months clinical and microbiological evaluation. Eur. J. Dent..

[22] Yaegaki K., Sanada K. (1992). Biochemical and clinical factors influencing oral malodor in periodontal patients. J. Periodontol..

[23] Miyazaki H., Sakao S., Katoh Y., Takehara T. (1995). Correlation between volatile sulphur compounds and certain oral health measurements in the general population. J. Periodontol..

[24] Van Tornout M., Dadamio J., Coucke W., Quirynen M. (2013). Tongue coating: Related factors. J. Clin. Periodontol..

[25] Seerangaiyan K., Jüch F., Winkel E.G. (2018). Tongue coating: Its characteristics and role in intra-oral halitosis and general health—A review. J. Breath Res..

[26] Koshimune S., Awano S., Gohara K., Kurihara E., Ansai T., Takehara T. (2003). Low salivary flow and volatile sulfur compounds in mouth air. Oral Surg. Oral Med. Oral Pathol. Oral Radiol..

[27] Decean L., Badea M., Rus V., Buicu G., Sasu A., Pilut C.N., Mihai A. (2022). The Implication of Misinformation and Stigma in Age-Related Quality of Life, Depression, and Coping Mechanisms of Adult Patients with Psoriasis. Medicina.

[28] Krämer S., Hillebrecht A.L., Wang Y., Badea M.-A., Barrios J.I., Danescu S., Fuentes I., Kartal D., Klausegger A., Ponce de León E. (2024). Orofacial Anomalies in Kindler Epidermolysis Bullosa. JAMA Dermatol..

[29] Stanciu I.-V., Fildan A.-P., Chenna V.S.H., Ilie A.C., Tudorache E., Rosca O., Stanga L., Cozma G.V., Preotesoiu I., Dantes E. (2025). Physiologic–Inflammatory–Nutrition (TRIAD-TB) Score at 72 Hours Predicts 30-Day Mortality and Length of Stay in Pulmonary Tuberculosis: A Prospective Cohort Study. Biomedicines.

[30] Braicu V., Stelian P., Fulger L., Verdes G., Brebu D., Duta C., Fizedean C., Ignuta F., Danila A.I., Cozma G.V. (2024). Impact of Systemic Treatments on Outcomes and Quality of Life in Patients with RAS-Positive Stage IV Colorectal Cancer: A Systematic Review. Diseases.

[31] Vaduva D.M.B., Velimirovici D.E., Vaduva M.M.B., Stanga L., Petrescu H., Rada M., Cipu D., Vaduva B.M.B., Radulescu M. (2018). Phenotypic Study and Sensitivity to Anti-Infective Chemotherapy of Bacterial Strains Isolated from Cutaneous-Mucosal Infections. Mater. Plast..

[32] Tapalaga G., Stanga L., Sîrbu I. (2025). Systematic Review of Lead Exposure and Its Effects on Caries and Aesthetics in Children and Adolescents. Healthcare.

[33] Stanciu I.-V., Fildan A.-P., Thakur B.R., Ilie A.C., Stanga L., Oancea C., Tudorache E., Bratosin F., Rosca O., Bogdan I. (2025). Full-Blood Inflammatory Ratios Predict Length of Stay but Not Early Death in Romanian Pulmonary Tuberculosis. Medicina.

[34] Barbu L.A., Vasile L., Cercelaru L., Șurlin V., Mogoantă S.-S., Mogoș G.F.R., Țenea Cojan T.S., Mărgăritescu N.-D., Buliman A. (2025). Non-Variceal Upper Gastrointestinal Bleeding: A Retrospective Cohort of 364 Cases, Historical Comparison, and Updated Management Algorithm. Life.

[35] Barbu L.A., Vasile L., Cercelaru L., Șurlin V., Mogoantă S.-Ș., Mogoș G.F.R., Țenea Cojan T.S., Mărgăritescu N.-D., Iordache M.P., Buliman A. (2025). Aggressiveness in Well-Differentiated Small Intestinal Neuroendocrine Tumors: A Rare Case and Narrative Literature Review. J. Clin. Med..

[36] Iordache M.P., Buliman A., Costea-Firan C., Gligore T.C.I., Cazacu I.S., Stoian M., Teoibaș-Şerban D., Blendea C.-D., Protosevici M.G.-I., Tanase C. (2025). Immunological and Inflammatory Biomarkers in the Prognosis, Prevention, and Treatment of Ischemic Stroke: A Review of a Decade of Advancement. Int. J. Mol. Sci..

[37] Blendea C.-D., Khan M.T., Stoian M., Gligore T.C.I., Cuculici Ș., Stanciu I.L., Protosevici M.G.-I., Iordache M., Buliman A., Costea-Firan C. (2025). Advances in Minimally Invasive Treatments for Prostate Cancer: A Review of the Role of Ultrasound Therapy and Laser Therapy. Balneo PRM Res. J..

[38] Buliman A., Calin M.A., Iordache M.P. (2025). Targeting Anxiety with Light: Mechanistic and Clinical Insights into Photobiomodulation Therapy: A Mini Narrative Review. Balneo PRM Res. J..

